# Strategies for increasing fertility in high productivity dairy herds

**DOI:** 10.21451/1984-3143-AR2018-0079

**Published:** 2018-08-17

**Authors:** Luiz Gustavo Bragança, Amanda F. Zangirolamo

**Affiliations:** 1 Neogen Reprodução Assistida, Ribeirão Preto, São Paulo, Brasil.; 2 National Institute of Science and Technology for Dairy Production Chain (INCT-LEITE), Universidade Estadual de Londrina, Rodovia Celso Garcia Cid - Campus Universitário, Londrina, Paraná, Brazil.; 3 Laboratório de Reprodução Animal, DCV-CCA-UEL, Londrina, Paraná, Brazil.

**Keywords:** dairy cow, heat stress, high productivity, management, reproduction

## Abstract

Over the years, the dairy cow has been selected and managed for high performance and efficiency in milk production. Thus, different factors influence the fertility of dairy cows of high productivity. It has been observed that genetic selection for large productions and metabolic adaptations may explain the greater requirement to maintain reproduction at satisfactory levels. Also, these animals are more susceptible to environmental factors such as increased temperature and humidity. Studies have shown that heat stress results in reduced estrous expression and impairs ovarian function, interfering with folliculogenesis and steroidogenesis. Likewise, under heat stress conditions, dry matter intake is reduced, prolonging the after calving negative energy balance and calving-conception interval. Thus, suboptimal reproductive performance is one of the main factors responsible for the economic losses in large dairy farms. In this context, numerous management practices have been introduced to improve reproduction in high productivity animals, making reproductive management increasingly complex in dairy farms. Among them, we can mention the implementation of management and nutrition conditions adapted to the periods of heat stress, as well as protocols and biotechniques that improve the quality of the follicles and oocytes. Thus, because genetic selection for better fertility animals is a characteristic of low heritability, the success of reproductive programs in highly productive herds depends on the association of the control of genetic and physiological factors with those of environmental, nutritional and management. This paper aims to discuss reproductive aspects of dairy cows of high productivity, the role of heat stress in this context, and the implementation of management, nutrition and biotechnology conditions, to minimize the adverse effects on the reproduction of these animals.

## Introduction

 The dairy industry in the world's major producing countries has changed drastically in the last decade. World milk production reached 811 million tonnes in 2017, 1.4 percent higher than in 2016 (Food and Agriculture Organization of the United Nations – FAO, 2018). The US leads the ranking with 93.5 thousand tons/year and the highest average productivity index per cow, 10,150 liters. Brazil, occupying the fourth place, from 1961 to 2015, presented an increase of 30 million tons in milk production in 54 years and an average annual gain of 555 thousand tons (FAO, 2016; Instituto Brasileiro de Geografia e Estatística – IBGE, 2016). In this scenario, there is a perspective of at least 47.5 million tons of milk by Brazilian industry in 2025 to serve the population of 219 million people (Ministério da Agricultura, Pecuária e Abastecimento – MAPA, 2015; Vilela, 2015).

 Milk production per cow has steadily increased through the combined improvement of management, nutrition, and genetic selection actions. However, farms are becoming larger and with more productive cows, increasing the challenge of maintaining reproductive efficiency in increasingly adverse situations due to metabolic adaptations for this purpose (Crowe *et al*., 2018). Thus, it is justified to maintain the most demanding reproduction at satisfactory levels, considering that high producing cows have a higher incidence of infertility or subfertility (Walsh *et al*., 2011). However, in addition to milk production, other factors are likely to decrease reproductive efficiency in these herds.

 The selection of cows to improve the genetic potential for high productivity also reduces the heat tolerance of dairy cows (Ravagnolo and Misztal, 2000). In this context, it has already been demonstrated that heat stress interferes with the expression of the signs of estrus and follicular diameter (Schüller *et al*., 2017), which may reduce conception rates, and high embryonic loss (Roche *et al*., 2011). Another effect of heat stress is the change in the patterns of ovarian follicular development and the gene expression in the oocyte (Ferreira *et al*., 2016). Likewise, it has also been described the reduction in dry matter intake (Dias *et al*., 2018), which in turn can prolong after calving negative energy balance and calving-conception interval.

 Finally, many researches have been carried out in the areas of genetics, physiology, nutrition, management, and applications of reproductive biotechnology in the last decade, to minimize the impact of high productivity on the reproductive rates of dairy cows. Thus, the success of reproductive programs in highly productive herds, due to the genetic selection for the best fertility animals, is a characteristic of low heritability, and it depends of the association of the practices and actions in all the areas mentioned above.

## Transition period and effects on reproduction

 In the transition period - three weeks prepartum up to three weeks after calving - there are major adaptive changes (physiological, metabolic and nutritional) characterizing the final period of gestation and the beginning of lactation. The way these changes occur and how they are managed are of great importance because they are closely linked to the performance of lactation, the occurrence of after calving diseases (clinical and subclinical) and reproductive efficiency, significantly affecting the profitability of the herds (Roche *et al*., 2018).

 The transition period, although short, is the phase during the productive cycle of lactating cows when most metabolic and infectious diseases occur, such as mastitis and metritis in the weeks immediately after birth, with implications for reproduction (Roche *et al*., 2013). Researchers have reported that the immune system of cows under metabolic stress is further reduced, demonstrating a relationship between metabolic status and peripartum immune function (Crookenden *et al*., 2017).

 Deep physiological changes occur in the cow in transition period, with drastic modifications in their metabolism. The rapid increase in fetal demands and the development of the mammary gland, including the initiation of the synthesis of milk components, cause these changes (Bell, 1995), reducing dry matter intake and inducing the mobilization of body stores of adipocytes (McArt *et al*., 2013).

 Such lipid mobilization increases the plasma concentration of non-esterified fatty acids that increase gradually in the prepartum period. A portion of this increase is mandatory and it is hormonally controlled while another part of this process is a result of an energy deficit (Dyk and Emery, 1996). This mechanism results in high concentrations of fatty acids circulating in the blood during the initiation of lactation. In this context, the high concentrations of fatty acids can lead to metabolic diseases such as hepatic lipidosis, and ketosis - a clinical condition caused by the accumulation of ketone bodies (intermediates in the breakdown of fatty acids) and defined by blood concentrations of b-hydroxybutyrate (Gordon *et al*., 2013).

 The occurrence of negative energy balance, high concentrations of fatty acids, b-hydroxybutyrate and triacylglycerol in the liver, coincide with the resumption of ovarian activity, development of follicles that supply oocytes for fertilization and uterine involution and remodeling (Roche *et al*., 2018). Thus, together, these processes and metabolic states can affect pre and post-ovulatory reproductive function (Luttgenau *et al*., 2016).

## Effects of heat stress on reproduction

 Cattle have specific thermoneutral zones, which are the environmental temperatures in which body heat production is in equilibrium with body heat loss, and thus, there is no need for additional warming or cooling mechanisms or behaviors. When the local temperatures exceed the threshold of the thermoneutral zone, it is possible to identify the heat stress (Brugemann *et al*., 2012).

 Studies have shown that hot climatic conditions are associated with reductions in both feed intake and milk production in dairy cows (Garner *et al*., 2017). Besides, the genetic selection process to improve milk production reduces heat tolerance (Ravagnolo and Misztal, 2000).

 In the reproductive context, heat stress interferes with the expression of the signs of estrus and follicular diameter (Schüller *et al*., 2017), which may reduce conception rates and may cause embryonic loss (Roche *et al*., 2011). In addition, this condition changes the gene expression patterns in the oocyte, retards follicular selection, and promotes adverse effects on steroidogenesis and oocyte quality (Roth *et al*., 2001 ; Ferreira *et al*., 2016).

 Heat stress is one of the main factors reducing the fertility of inseminated dairy cows during the hot months of the year (Al-Katanani *et al*., 1999). There are clear seasonal differences in oestrus detection, conception rate, and maintenance of gestation between the summer and winter months (De Rensis *et al*., 2002). The uterine environment is also compromised by decreased blood flow and elevated temperature, and these changes impair embryonic development and increase the incidence of embryonic death (Rivera and Hansen 2001).

 Likewise, it has also been described that the reduction in dry matter intake (Dias *et al*., 2018), can prolong after calving negative energy balance and calving-conception interval. The reduction in dry matter intake and the prolongation of the negative energy balance promotes a decrease in plasma insulin, glucose, and IGF-I concentrations and increases the plasma concentration of growth hormone (GH) and non-esterified fatty acids. These changes in metabolic hormones act negatively on the hypothalamic-pituitary axis and the ovaries, mediating the inhibitory effects of negative energy balance on after calving fertility (De Rensis and Scaramuzzi, 2003).

**Figure 1:**
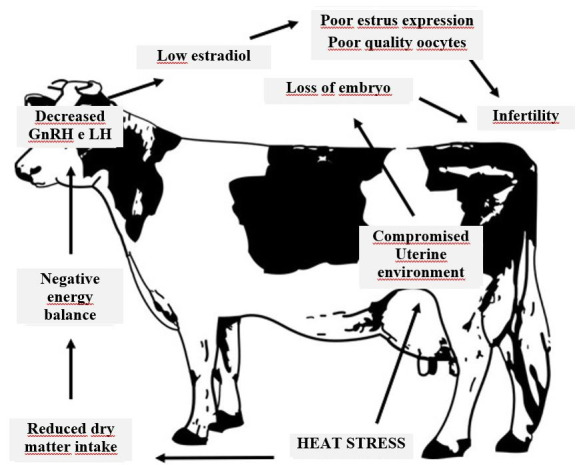
A schematic description of the possible mechanisms for the effect of heat stress on reproduction in the lactating dairy cow. Adapted from: De Rensis and Scaramuzzi (2003) .

 In conclusion, heat stress has a wide range of effects on the reproductive axis. Some of these effects directly affect individual reproductive organs such as the hypothalamus, anterior pituitary, follicle, oocyte, and embryo, while other effects are indirect and probably mediated by changes in the metabolic axis in response to reduction of dry matter intake (Fig.1).

## Strategies for increasing fertility in dairy cows

### Alternatives to minimize the effects of the transition period and heat stress

 Diseases in early lactation have a profound impact on the fertility of dairy cows. Thus, the maintenance of metabolic health minimizes the risks of after calving clinical and subclinical problems benefiting reproduction (Santos and Ribeiro, 2014). Therefore, actions such as reducing after calving negative energy balance to ensure that concentrations of fatty acid and b-hydroxybutyrate in the blood are controlled (Raboisson *et al*., 2014), will probably increase reproductive success.

 Among the practices that can be used to minimize the effects of heat stress in dairy cows are, for example, regarding temperature and humidity control, vitamin and mineral supplementation, as well as the transfer of embryos – instead of artificial insemination – in strategic (hot) days for such situation.

 About temperature and humidity control actions, there are basically two methods for cooling lactating cows. The first one is to modify the environment to prevent or avoid the degree of thermal stress that the cows are exposed and the second action is to improve the heat exchange between the cow and its environment (Amaral *et al*., 2009). In practice, this means to increase the rate of evaporative cooling by wetting cows or surrounding them through sprinklers or nebulizers and increasing the rate of convective heat transfer (increased air velocity over cows) through, for example, ventilators (Renaudeau *et al*., 2012).

 Shading of feed and water also offers dairy production advantages. Among the several methods already reported to reduce heat stress in cows, simple shade materials appear to be the most cost-effective methods (Kamal *et al*., 2018). Different materials are being used to provide shade during hot weather. However, in choosing the material shade to be used, it must be observed that it is weatherproof, strong, durable, poor heat conductor and that it prevents condensation of moisture inside (Kamal *et al*., 2016).

 The heat stress has also been associated with reduced total antioxidant activity in blood plasma (Sordillo and Aitken, 2009). There is some evidence that depression in embryonic survival after exposure to elevated temperatures involves increased production of free radicals. Thus, dietary supplements like vitamins, trace elements, and minerals are used to minimize the unfavorable effects of thermal stress. Khorsandi *et al*. (2016)  reported that supplementation of transition cows under heat stress with ruminal bolus led to increased milk production, attenuating the adverse effects caused by these conditions. In this context, zinc supplementation, described by mitigating the heat shock protein response and enhancing immunity in animals, may, for example, be further explored to decrease the adverse effects of heat stress in dairy cows (Sheikh *et al*., 2017).

 In cattle, the efficiency of production of embryos using superovulation protocols is impaired in periods when there is the highest ambient temperature. Climatic variations at the beginning of the period of activation of ovarian follicular development up to the time of fertilization may interfere with the production of embryos. Thus, actions such as changing the time of embryo transfer protocols, based on previous and current climate patterns, seems to be an alternative to optimize the return on investment when using these reproductive technologies (Chinchilla-Vargas *et al*. al. 2018).

 However, although the use of these systems and actions may significantly improve the fertility of lactating cows during the summer, it is desirable to consider a global view on the process, avoiding isolated practices.

### Use of reproductive biotechniques

 The herd of dairy cows has been selected and managed for high yield and efficiency (Roche *et al*., 2018). Thus, the findings of physiological mechanisms made early in the last century provided insights for the development and enhancement of technologies and programs used today to control reproduction in dairy cattle.

 The reproductive efficiency of dairy herds is usually low. Part of this inefficiency is due to factors resulting from high milk production. However, most of this is due to the low service rate. Cows that become pregnant after 120 days of lactation contribute to the increase in the interval between calving of the herd, with the reduction of average daily production and consequently with less profit. With the advent of fixed-time insemination (FTAI) protocols that synchronize follicular growth, regression of the corpus luteum and ovulation, a rate of 100% of inseminated animals can be achieved without the need for estrus observation (Colazo and Mapletoft, 2014). Increasing the service rate without relying on human failures to detect estrus, it becomes a great strategic tool to improve service rates and reproductive rates.

 There are fundamental points for the success of the reproductive programs in dairy herds of high productivity. For instance, the number of days after delivery to the first artificial insemination; the moment of pregnancy diagnosis; and the strategies for cows after being diagnosed as non-pregnant..

 The first step is to determine how many days after calving (days in milk) the cows will receive the first AI and the second step is to monitor if the planning is running. Usually, the herds define a voluntary waiting period that varies between 45 and 55 days after calving to begin breeding cows. There are also other factors, such as the choice of the reproductive program, the proper management of the cows during the pre and after calving period and the use of specific farm management softwares.

 The use of ultrasonography is very important to reproductive efficiency. Using this method, the diagnosis of pregnancy is usually determined from day 28 of pregnancy, and 30 days later it is usually performed to evaluate embryonic loss. Another useful strategy is to resynchronize cows after unsuccessful insemination (Crowe *et al*., 2018).

 Finally, over the past 40 years, bovine IVF has evolved from an experimental procedure for the treatment of infertile animals to a genomic accelerator for many breeds. In this scenario, genomic selection of young animals and embryos combined with sexing technologies and new freezing methods are driving a new era of *in vitro* fertilization in the global dairy industry. Thus, the progress in the use of this biotechnology has enabled the production of millions of valuable animals (Sirard, 2018).

## Conclusion

 The success of reproductive programs for high producing dairy cows goes beyond simply choosing the reproductive biotechniques to be used. Especially in Brazil, much of the low reproductive efficiency is due to the long period of heat stress that the cows are exposed.

 In short, because genetic selection for better fertility animals is a characteristic of low heritability, the success of reproductive programs in high productivity dairy herds depends on the combination of the use of reproductive biotechniques associated with management and nutrition practices that minimize the effects caused by heat stress and transition period.

## References

[B001] Al-Katanani YM, Webb DW, Hansen PJ (1999). Factors affecting seasonal variation in non return rate to first service in lactating Holstein cow in a hot climate. *J Dairy Sci*.

[B002] Amaral BC, Tao S, Hayen J, Connor EE, Bubolz J, Dahl GE (2009). Heat stress abatement during the dry period: does cooling improve transition into lactation?. *J Dairy Sci*.

[B003] Bell AW (1995). Regulation of organic nutrient metabolism during transition from late pregnancy to early lactation. *J Anim Sci*.

[B004] Brugemann K, Gernand E, Konig von Borstel U, Konig S. (2012). Defining and evaluating heat stress thresholds in different dairy cow production systems. *Archiv fur Tierzucht*.

[B005] Chinchilla-Vargas J, Jahnke MM, Dohlman TM, Rothschild MF, Gunn PJ (2018). Climatic factors affecting quantity and quality grade of *in vivo* derived embryos of cattle. *Anim Reprod Sci*.

[B006] Colazo MG, Mapletoft RJ (2014). A review of current timed-AI (TAI) programs for beef and dairy cattle. *Can Vet J*.

[B007] Crookenden MA, Walker CG, Heiser A, Murray A, Dukkipati VSR, Kay JK, Meier S, Moyes KM, Mitchell MD, Loor JJ, Roche JR (2017). Effects of precalving body condition and prepartum feeding level on gene expression in circulating neutrophils. *J Dairy Sci*.

[B008] Crowe MA, Hostens M, Opsomer G (2018). Reproductive management in dairy cows - the future. *Ir Vet J*.

[B009] De Rensis F, Marconi P, Capelli T, Gatti F, Facciolongo F, Franzini S (2002). Fertility in postpartum dairy cows in winter or summer following estrous synchronization and fixed time A.I. after the induction of an LH surge with Gonadotropin releasing hormone (GnRH) or human chorionic gonadotropin (hCG). *Theriogenology*.

[B010] De Rensis F, Scaramuzzi RJ (2003). Heat stress and seasonal effects on reproduction in the dairy cow-a review. *Theriogenology*.

[B011] Dias JDL, Silva RB, Fernandes T, Barbosa EF, Graças LEC, Araujo RC, Pereira RAN, Pereira MN (2018). Yeast culture increased plasma niacin concentration, evaporative heat loss, and feed efficiency of dairy cows in a hot environment. *J Dairy Sci*.

[B012] Dyk P, Emery R (1996). Reducing the incidence of peripartum health problems..

[B013] Ferreira RM, Chiaratti MR, Macabelli CH, Rodrigues CA, Ferraz ML, Watanabe YF, Smith LC, Meirelles FV, Baruselli PS (2016). The infertility of repeat-breeder cows during summer is associated with decreased mitochondrial DNA and increased expression of mitochondrial and apoptotic genes in oocytes. *Biology of Reproduction*.

[B014] (FAO) (2016). Division, trade, downloads data, crops and livestock products.

[B015] (FAO) (2018). Dairy Market Review.

[B016] Garner JB, Douglas M, Williams SRO, Wales WJ, Marett LC, DiGiacomo K, Leury BJ, Hayes BJ (2017). Responses of dairy cows to short-term heat stress in controlled-climate chambers. *Animal Prod Sci*.

[B017] Gordon JL, LeBlanc SJ, Duffield TF (2013). Ketosis treatment in lactating dairy cattle. *Vet Clin North Am Food Anim Pract*.

[B018] (IBGE) (2016). Pesquisa da pecuária municipal e censo agropecuário.

[B019] Kamal R, Dutt T, Patel BHM, Singh G, Chandran PC, Dey A, Barari SK (2016). Effect of shade materials on rectal temperature, respiration rate and body surface temperature of crossbred calves during rainy season. *Indian J Anim Sci*.

[B020] Kamal R, Dutt T, Patel M, Dey A, Bharti PK, Chandran PC (2018). Heat stress and effect of shade materials on hormonal and behavior response of dairy cattle: a review. *Trop Anim Health Prod*.

[B021] Khorsandi S, Riasi A, Khorvash M, Mahyari SA, Panah FM, Ahmadi F (2016). Lactation and reproductive performance of high producing dairy cows given sustained- release multi-trace element/vitamin ruminal bolus under heat stress condition. *Livest Sci*.

[B022] Luttgenau J, Purschke S, Tsousis G, Bruckmaier RM, Bollwein H (2016). Body condition loss and increased serum levels of nonesterified fatty acids enhance progesterone levels at estrus and reduce estrous activity and insemination rates in postpartum dairy cows. *Theriogenology*.

[B023] McArt JAA, Nydam DV, Oetzel GR, Overton TR, Ospina PA (2013). Elevated non-esterified fatty acids and b-hydroxybutyrate and their association with transition dairy cow performance. *Vet J*.

[B024] (MAPA) (2015). Projeções do Agronegócio: Brasil 2014/2015 a 2024/2025 / Ministério da Agricultura, Pecuária e Abastecimento.

[B025] Raboisson D, Mounie M, Maigne E (2014). Diseases, reproductive performance, and changes in milk production associated with subclinical ketosis in dairy cows: a meta-analysis and review. *J Dairy Sci*.

[B026] Ravagnolo O, Misztal I (2000). Genetic component of heat stress in dairy cattle, parameter estimation. *J Dairy Sci*.

[B027] Renaudeau D, Collin A, Yahav S, de Basilio V, Gourdine JL, Collier RJ (2012). Adaptation to hot climate and strategies to alleviate heat stress in livestock production. *Animal*.

[B028] Rivera RM, Hansen PJ (2001). Development of cultured bovine embryos after exposure to high temperatures in the physiological range. *Reproduction*.

[B029] Roche JR, Burke CR, Meier S, Walker CG (2011). Nutrition reproduction interaction in pasture-based systems: is nutrition a factor in reproductive failure?. *Anim Prod Sci*.

[B030] Roche JR, Bell AW, Overton TR, Loor JJ (2013). Nutritional management of the transition cow in the 21^st^ century – a paradigm shift in thinking. *Anim Prod Sci*.

[B031] Roche JR, Burke CR, Crookenden MA, Heiser A, Loor JL, Meier S, Mitchell MD, Phyn CVC, Turner SA (2018). Fertility and the transition dairy cow. *Reprod Fertil Dev*.

[B032] Roth Z, Meweidan R, Shaham-Albalancy A, Braw-Tal R, Wolfenson D (2001). Delayed effect of heat stress on steroid production in medium-size and preovulatory bovine follicles. *Reproduction*.

[B033] Santos JEP, Ribeiro ES (2014). Impact of animal health on reproduction of dairy cows. *Anim Reprod*.

[B034] Schüller LK, Michaelis I, Heuwieser W (2017). Impact of heat stress on estrus expression and follicle size in estrus under field conditions in dairy cows. *Theriogenology*.

[B035] Sheikh AA, Aggarwal A, Indu B, Aarif O (2017). Inorganic zinc supplementation modulates heat shock and immune response in heat stressed peripheral blood mononuclear cells of periparturient dairy cows. *Theriogenology*.

[B036] Sirard MA (2018). 40 years of bovine IVF in the new genomic selection context. *Reproduction*.

[B037] Sordillo LM, Aitken SL (2009). Impact of oxidative stress on the health and immune function of dairy cattle. *Vet Immunol Immunopathol*.

[B038] Vilela D. (2015). Para onde caminha o leite. *Rev Balde Branco*.

[B039] Walsh SW, Williams EJ, Evans ACO (2011). A review of the causes of poor fertility in high milk producing dairy cows. *Anim Reprod Sci*.

